# The Impact of Neoadjuvant Chemotherapy on Ovarian Cancer Tumor Microenvironment: A Systematic Review of the Literature

**DOI:** 10.3390/ijms25137070

**Published:** 2024-06-27

**Authors:** Giulia Spagnol, Eleonora Ghisoni, Matteo Morotti, Orazio De Tommasi, Matteo Marchetti, Sofia Bigardi, Valentina Tuninetti, Giulia Tasca, Marco Noventa, Carlo Saccardi, Roberto Tozzi, Denarda Dangaj Laniti

**Affiliations:** 1 Unit of Gynecology and Obstetrics, Department of Women and Children’s Health, University of Padua, 35122 Padua, Italy; 2 Department of Oncology, Lausanne University Hospital, University of Lausanne (UNIL), 1015 Lausanne, Switzerland; 3 Lausanne Branch, Ludwig Institute for Cancer Research, University of Lausanne (UNIL), 1015 Lausanne, Switzerland; 4 Agora Cancer Research Center, 1005 Lausanne, Switzerland; 5 Department of Oncology, Ordine Mauriziano Hospital, University of Turin, 10124 Turin, Italy; 6 Istituto Oncologico Veneto IOV-IRCCS, 35128 Padova, Italy

**Keywords:** ovarian cancer, NACT, TILs

## Abstract

Immunotherapy, particularly the use of immune checkpoint inhibitors (ICIs), has shown limited efficacy in treating ovarian cancer (OC), possibly due to diverse T cell infiltration patterns in the tumor microenvironment. This review explores how neoadjuvant chemotherapy (NACT) impacts the immune landscape of OC, focusing on tumor-infiltrating lymphocytes (TILs), PD-1/PD-L1 expression, and their clinical implications. A comprehensive literature search across four databases yielded nine relevant studies. These studies evaluated stromal (sTILs) and intra-epithelial (ieTILs) TILs before and after NACT. sTIL responses varied, impacting prognostic outcomes, and ieTILs increased in some patients without clear survival associations. PD-L1 expression after NACT correlated with improved overall survival (OS), and increases in granzyme B+ and PD-1 correlated with longer progression-free survival (PFS). Remarkably, reduced FoxP3+ TILs post-NACT correlated with better prognosis. NACT often increases sTIL/ieTIL and CD8+ subpopulations, but their correlation with improved PFS and OS varies. Upregulation of co-inhibitory molecules, notably PD-L1, suggests an immunosuppressive response to chemotherapy. Ongoing trials exploring neoadjuvant ICIs and chemotherapy offer promise for advancing OC treatment. Standardized measurements assessing TIL density, location, and heterogeneity are crucial for addressing genetic complexity and immunological heterogeneity in OC.

## 1. Introduction

Ovarian cancer (OC) is the most lethal gynecologic malignancy, with a 5-year overall survival of approximately 40–45% [1,2]. Standard first-line treatment for patients with advanced-stage OC consists of cytoreductive surgery, either primary debulking surgery (PDS) or interval debulking surgery (IDS), after neoadjuvant chemotherapy (NACT) [3] and platinum-/taxane-based chemotherapy. However, the majority of patients will eventually experience relapse and become increasingly resistant to chemotherapy, historically defined as “platinum resistance”. Resistance to platinum-based treatment is common, with roughly 20% of women experiencing disease progression ≤6 months after completing a platinum-based regimen (classified as “platinum-resistant” relapse) or who fail to respond at all to first-line treatment or relapse within 4–6 weeks after last platinum dose (classified as “platinum-refractory”). Life expectancy for platinum-resistant patients does not exceed one year, and new treatment options are urgently needed to significantly increase their response rate and survival. The recent development of poly (ADP-ribose) polymerase (PARP) inhibitors has reopened the possibility of making a significant impact on OC outcomes in both BRCA mutant and BRCA wild-type tumors [4]. The pioneering study was a SOLO-1 trial demonstrating that the use of Olaparib in advanced-stage OC patients with BRCA1/2 mutations led to a significant prolongation of progression-free survival (PFS) [5]. More recently, the PRIMA trial showed benefits of Niraparib even in patients without BRCA mutations, suggesting the effectiveness of these drugs in the absence of homologous recombination deficiency [6].

Immunotherapy using immune checkpoint inhibitors (ICIs) that mainly act on the programmed cell death-1 and its ligand (PD1/PD-L1) immune axis have been introduced as the standard of care for a variety of cancers including melanoma [7], Hodgkin’s lymphoma [8], non-small-cell lung cancer [9], renal cell carcinomas, and bladder cancer [10].

Although OC is in principle immunoreactive, as demonstrated by the significant expression of a variety of tumor-associated antigens [11] and the evidence that half of treatment-naive adnexal tumors are infiltrated by intraepithelial tumor-infiltrating lymphocytes (TILs) that correlate with improved survival [12,13,14,15,16], ICIs have fallen short of expectations in OC so far. Responses to PD1 or PD-L1 blockade have resulted in an average response rate of 10 to 15% and a disease control (complete response or partial response) observed in less than half of the patients [17].

The notion of intratumoral versus intraepithelial patterns of T cell infiltration in the tumor microenvironment (TME) drew attention to the importance of lymphocyte infiltration in human tumors and inspired a working classification of all solid tumors into three immune phenotypes: (1) cold ones (also named desert or non-infiltrated), in which T cells are either absent or present in very low numbers; (2) the excluded, in which T cells accumulate in the tumor stroma; and (3) hot tumors (also named inflamed), in which T cells infiltrate the tumor epithelium [18,19].

T cell-inflamed tumors should, in theory, be suitable candidates for ICI therapy, as their TME is already conducive to immune attack [20,21]. Thus, the immune classification could provide an easily accessible in situ tissue biomarker for patient selection. However, the significance and evolution of TIL infiltration in OC patients undergoing NACT is poorly understood.

In this review, we aimed to provide an update on the effect of NACT on OC TME with regard to TIL infiltration (stromal versus intraepithelial), PD1/PD-L1 expression, and other immune cell populations, as well as their clinical prognostic implications.

## 2. Methods

### 2.1. Information Sources and Search Strategies

This systematic review was conducted according to the Preferred Reporting Items for Systematic reviews and Meta-Analysis (PRISMA) guidelines. A literature search of relevant papers was conducted using the following electronic bibliographic databases: Medline, Scopus, Embase, Science Direct, the Cochrane Library, Clinicaltrials.gov, the Cochrane Central Register of Controlled Trials, the EU Clinical Trials Register, and the World Health Organization International Clinical Trials Registry Platform. The strategies and keywords for electronic searching were the following: ovarian cancer, tumor-infiltrating lymphocytes (TILs), NACT, stromal tumor-infiltrating lymphocytes (sTILs), immunotherapy, immune checkpoint, immune cell subpopulations, intraepithelial tumor-infiltrating lymphocytes (ieTILs), PD-1, and PD-L1.

### 2.2. Eligibility Criteria

We included in this systematic review all types of observational and/or descriptive studies that evaluated TIL infiltrations, PD-1 and PD-L1 expression, and other immune cell populations in ovarian cancer tissue specimens before and after NACT. Narrative or systematic reviews and case reports were excluded from the analysis. TIL infiltration was defined as the percentage of total stromal or intraepithelial area occupied by mononuclear cells according to the method described by the International TILs Working Group 2014 [22].

The inclusion criteria applied in this systematic review were the following:

- Type of study: case-control studies, cohort studies, or case series.
- Period of publication: no restriction.
- Language: only English-language studies were included.
- Data search: January 2003–December 2023.
- Participants: women with ovarian cancer who underwent NACT with available samples before and after chemotherapy, as well as immune cell infiltration evaluation.
- Comparators: (i) sTILs and ieTILs before and after NACT; (ii) PD-1, PD-L1 expression and other immune cell populations before and after NACT.
- Outcomes: prognostic impact of sTILs and ieTILs, PD-1/PD-L1, and other immune cell populations before and after NACT in terms of PFS and OS.

### 2.3. Study Selection and Data Extraction 

Titles and/or abstracts of studies retrieved using the electronic search strategy described above were screened independently by two review authors (G.S., E.G.) to identify studies that potentially met the inclusion criteria. Any disagreement over the eligibility of a study was resolved through discussion with a third external collaborator (M.M.). Then, the full text of potentially eligible studies was retrieved and independently assessed for final eligibility. We extracted data about study characteristics (design and time of the study), study population (number and characteristics of enrolled patients), methods of analysis of cell infiltration (immunohistochemistry [IHC], multiplex immunofluorescence [mIF]), type of immune cell infiltration evaluated (ie CD4+, CD8+, Foxp3+, PD-1+, PD-L1+, …), study conclusions in terms of clinical impact (predictive and/or prognostic) PFS, and OS.

A manual search of the reference list of included studies was also performed to avoid missing relevant data. We searched for published (full-text studies and meeting abstracts) studies from the aforementioned electronic databases. The results were compared, and any disagreement was resolved by consensus.

## 3. Results and Discussion

### 3.1. Study Selection

A total of 475 relevant titles were identified from the literature search of Medline, Embase, Science Direct, the Cochrane Central Register of Controlled Trials, and the Cochrane Database of Systematic Reviews.

We excluded 265 duplicates. Among the 210 records, 176 were removed during title and abstract screening, leaving 34 articles eligible for full-text review.

**Figure 1:**
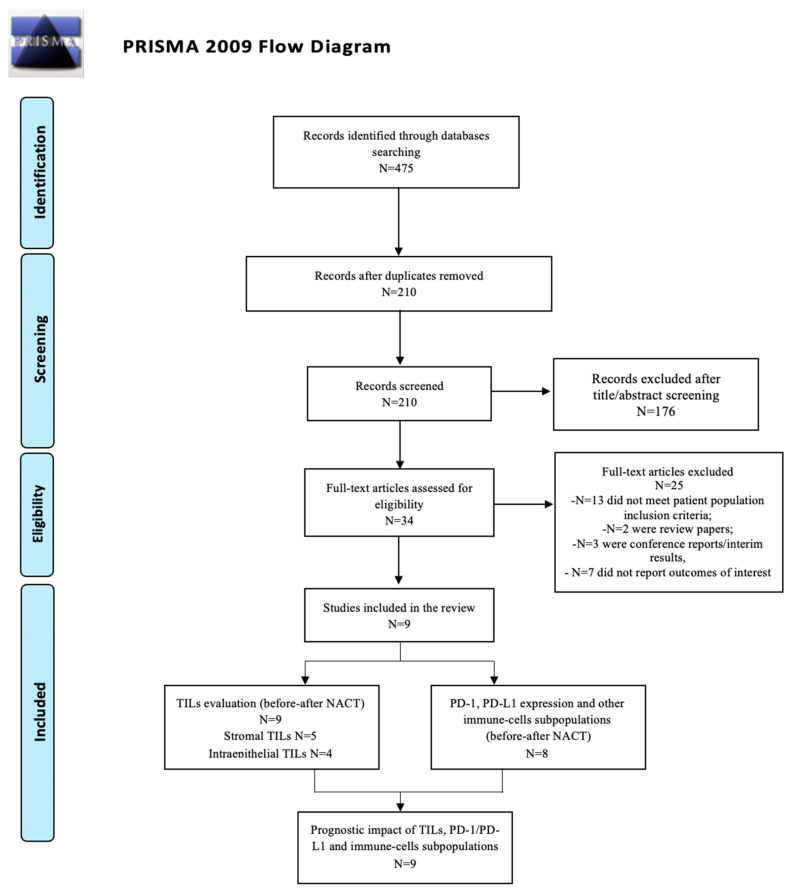
PRISMA flow diagram.

Of these, 25 were deemed ineligible due to the following reasons: 13 did not meet inclusion criteria in terms of patient population, 2 were review papers, 3 were conference proceedings/interim analysis, and 7 did not report outcomes of interest (PFS and/or OS). Finally, nine studies met all the criteria for the inclusion in this systematic review [23,24,25,26,27,28,29,30,31]. The PRISMA flow diagram of the study selection process is shown in Figure 1. Among the nine included studies, eight were retrospective, and one was a prospective study (see Table 1 for extended details). Included studies were published between 2003 and 2022.

#### Study and Patient Characteristics Overview

The first study that evaluated the possible types of immune cell infiltration before and after NACT and its impact on PFS and OS was published by Polcher et al. in 2010 [23]. The authors examined the associations between the extent of ieTILs, CD4+ and CD8+ T cells, Foxp3+ and granzyme B+ intraepithelial infiltration, as well as the ratio between CD8+/CD4+ T cells, CD4+/Foxp3+ and CD8+/Foxp3+, on samples obtained from 30 patients with intraperitoneal biopsies collected prior to NACT and at IDS. In 2016, Bohm et al. assessed patient-matched omental biopsies obtained both at the time of diagnosis and during IDS from a cohort of 54 patients [24]. The biopsies were stained for CD8+ T cells and Foxp3+ Treg cells. In the same year, Lo et al. evaluated a broader range of immune cells (CD3+, CD8+, CD20+, CD68+, PD-1+, Foxp3+, and PD-L1+) on two different cohorts: one including 26 cases of matched pre- and post-NACT tumor samples and a second expanded cohort that included 64 cases for which only post-NACT samples were available [25].

In 2017, Mesnage et al. evaluated sTILs, ieTILs, and PD-L1, respectively, in 83, 76, and 27 patients with paired pre- and post-NACT samples [26]. The assessment of tumor-infiltrating lymphocytes (TILs) was conducted using hematoxylin and eosin staining. Moreover, the authors proposed for the first time a new score for both ieTIL and sTIL assessment: For ieTILs, defined as lymphocytes in direct contact with the epithelium of tumor cell islets, a semi-quantitative assessment of infiltration yielded a score of 0–3 (0. no infiltration, 1. mild, 2. moderate, or 3. strong). High sTILs were defined as 50% infiltration, and high ieTILs were defined as a score ≥ 2. Similarly, Kim et al. performed immunostaining to evaluate the expression of PD-L1 in tumors (n = 37 matched samples pre- and post-NACT) and in stroma (n = 34) [27]. Chen et al. analyzed the relationship between PD-L1 expression and NACT in 15 patients post-NACT and 72 patients in treatment-naive tumors [28]. A large study by Leray et al. analyzed CD3+, CD8+, Foxp3+, CD68+, and CD4+ infiltration in 145 pre-NACT and 139 post-NACT samples; however, variations in the count of assessable paired samples for each marker were observed due to the presence of crush artifacts [29]. Finally, Félix Blanc-Durand et al. included 98 patients for a total of 173 tumor samples (77 taken at diagnosis, 77 post-NACT, and 19 at relapse). In particular, a total of 63 paired samples were available for the evaluation of IDO, PDL1, LAG3, and TIM3 ligands [30]. More recently, Lee et al. compared 147 paired samples evaluating CD8+, PD-L1+, and Foxp3+. Detailed extensive data are reported in Table 1 [31].

### 3.2. Tumor-Infiltrating Lymphocytes (TILs)

#### 3.2.1. Stromal TILs (sTILs)

Two studies evaluated sTIL infiltration without distinguishing further immune-cells subpopulations (Table 2).

In 2017, Mesnage et al. found an overall significant increase in the level of sTILs following NACT (median sTILs were 20% before to 30% after NACT, *p* = 0.0005) [26]. However, individual patients’ responses to NACT were highly variable. In particular, in this paper, we found three patterns of response (increasing, stable, and decreasing), with 51% of patients (42/83) showing an increase, 25% of patients (21/83) showing a decrease, and 24% (20/83) showing a stable level of sTILs upon treatment. Analyzing the prognostic impact, the authors found that both at univariate and multivariate analysis, higher levels of pre- and post-NACT sTILs had a significant beneficial prognostic effect on PFS whether analyzed as a continuous or categorical variable; on the contrary, multivariate continuous analysis of post-NACT sTILs did not reach significance (*p* = 0.10). The level of pre-NACT sTILs was not predictive of first PFI (platinum-free interval). High post-NACT sTILs, on the other hand, predicted subsequent platinum responsiveness (PFI ≥ 6 months). Furthermore, both high pre- and post-NACT sTILs were independent PFS predictors. Changes in sTILs were assessed in paired samples and defined as an absolute change of ≥10% or change in score ≥ 1, respectively. Indeed, there was a trend toward improved OS for patients with high levels of post-NACT sTILs, but not for patients with high levels of pre-NACT sTILs [26].

The same results finding an overall increase in sTILs after NACT were confirmed by Kim et al. The median levels were 5% and 10% in pre- and post-NAC samples, respectively, with high variability among individual patients. Of 34 patients available for assessment, in eight (23.5%) cases, stromal TILs remained at a similar level, but in nine (26.5%) cases, a decreased stromal TIL level was noted [27]. In most of the samples (17 (50%)), an increase in sTILs upon NACT was shown. However, the authors found that high pre-NACT sTILs assessed as proposed by Mesnage et al. [26] did not show any significant impact on either PFS (*p* = 0.899) or OS (*p* = 0.539) [27].

Three studies evaluated the sTIL subpopulation more in depth. One of the first ones was published by Bohm et al. [24]. The biopsies were stained for CD8+ T cells, CD45RO+ memory cells, and Foxp3+ Treg cells, and the results were compared to the chemotherapy response score (CRS). The authors found no association in the density of cells positive for the above markers before NACT between CRS1/2 (poor responders) and CRS3 responders (good responders). After NACT, there were still marked infiltrates of CD8+ T cells and CD45RO+ memory cells in the stroma and again no difference in the density of those populations between CRS1/2 or CRS3 subgroups. Furthermore, they observed that CD8+ cell density increased in approximately 50% of patients, but the tumor/stroma ratio of CD8+ T cells and CD45RO+ was unchanged before and after NACT. In contrast, there was a significant decline in the density of Foxp3+ cells in the stromal areas of the biopsies after NACT (*p* = 0.02). Good responder patients with samples scored as CRS3 had significantly improved PFS (*p* = 0.002) and OS (*p* = 0.03) compared with “poor responder” patients whose biopsies scored as CRS2, possibly related to the increase in CD8+ cell density [24].

Leary et al. also showed that stromal CD3+ T cells increased significantly after NACT (*p* = 0.003 in overall patients; *p* = 0.02 in paired samples), but again, the impact of NACT was clearly variable among individual patients (55% of the patients showing an increase, 37% showing a decrease, and 8% showing no change), with similar patterns observed for stromal CD8+ T cell infiltration (*p* = 0.001 in overall patients; *p* = 0.008 in paired samples) [29]. No significant difference was observed in median stromal CD4+ and Foxp3+ score before and after NACT. Given the heterogeneous patterns of change in sTIL subpopulations, the authors further evaluated the ratios of CD8+/Foxp3+, CD4+/Foxp3+, and CD3+/Foxp3+. Only the median of CD8+/Foxp3+ ratios increased significantly after NACT (*p* = 0.0001). Similar to other studies, none of the stromal T-cell subsets in pre-NACT samples correlated with PFS. The impact of immune infiltration levels after NACT on PFS and OS was then investigated: high stromal FOXP3 was the only immune marker associated with PFS (*p* = 0.03) but not OS, and neither CD3+, CD8+, or CD4+ TILs showed any significant correlation with PFS or OS after NACT.

More recently, Lee et al. found that in 17.1% of OCs, the initially low sTIL level increased to a high level after NACT (*p* = 0.001) [31]. Furthermore, they assessed the correlation between changes in CD8+ and Foxp3+ and the TIL level in matched samples before and after NACT: both CD8+ T cells and Foxp3+ T cells were positively correlated with increasing TILs (CD8+: *p* = 0.002; Foxp3+: *p* = 0.003). A high level of sTILs pre-NACT was associated with a significantly improved PFS (*p* = 0.039) and a better OS trend (*p* = 0.077). However, the PFS (*p* = 0.01) and OS (*p* = 0.027) were significantly lower in patients with increased TIL density after NACT [31].

#### 3.2.2. Intraepithelial TILs (ieTILs)

One study evaluated ieTIL infiltration without distinguishing further immune cell subpopulations (Table 3).

The paper by Mesnage et al. evaluated ieTILs alone, showing that ieTILs increased following NACT in 33% of patients, remained stable in 43%, and decreased in 24% of patients, respectively. Thirty-one percent of low-ieTIL tumors converted to high with NACT, and fifty-nine percent converted from high to low ieTILs. Differently to sTILs, ieTILs were not found to be associated with survival [26].

Three studies further evaluated the ieTIL subpopulations.

In 2010, Polcher et al. described that the mean number of infiltrating CD4+ and CD8+ cells was significantly enhanced after NACT as compared to baseline (CD4+: 2.5-fold increase, CD8+: 2.5-fold), whereas Foxp3+ cell numbers were equivalent before and after NACT samples [23]. In addition, CD8+/Foxp3+ cell ratios were significantly elevated after NACT (2.6-fold increase), whereas CD8+/CD4+ remained unaffected by chemotherapy. The authors discovered that none of the T cell infiltrates examined were associated with improved clinical outcome in pre-NACT specimens: TILs were prognostically neutral for both PFS and OS before NACT. Instead, low Foxp3+ cell density (<3.75/HPF) after NACT was associated with longer PFS, as compared with strong infiltration (median 20.94 vs. 11.24 months), and with improved OS (median 30.75 vs. 16.04 months). In addition, whereas CD8+/CD4+ T cell ratio prior to NACT had no influence on either PFS or OS, high CD8+/CD4+ T cell ratio after NACT (>1.96) was predictive of improved OS (median 62.10 vs. 20.74 months, respectively). The authors concluded that NACT elicited an immunologic profile in which low immunosuppressive Foxp3+ infiltration was significantly associated with PFS, suggesting a contribution of immunologic effects to improved clinical outcome [23].

Some years later, Lo et al. evaluated 26 OC cases for which pre- and post-NACT samples were available to quantify TIL densities [25]. In pre-NACT samples, CD3+ TILs were found in all cases but at widely varying densities (1.9–61.6 cells/100 tumor cells). Similar results were seen for both CD4+ TILs (0.1–18.9 cells/100 tumor cells) and CD8+ TILs (0.4–29.3 cells/100 tumor cells). In contrast, CD20+ TILs were found in fewer cases (77%) and at much lower densities (0–1.6 cells/100 tumor cells). Following NACT, the intraepithelial density of CD3+ T cells increased from a median of 7.0 to 18.6 cells/100 tumor cells (*p* = 0.013). Significant increases were also seen for CD8+ TILs, from a median of 6.7 to 13.5 cells/100 tumor cells (*p* = 0.006), and CD20+ TILs, from a median of 0.2 to 1.0 cells/100 tumor cells (*p* = 0.006). There was also a trend toward increased density of CD4+ T cells, from a median of 6.4 to 9.8 cells/100 tumor cells (*p* = 0.060). Thus, NACT was associated with a selective increase in the density of intraepithelial T cells (primarily the CD8 subset) and CD20+ B cells [25].

The authors further interrogated the clinical outcomes in an expanded cohort (n = 90) with similar clinicopathologic characteristics to the initial cohort, for which only post-NACT samples were available. Effector markers (e.g., CD4, CD8, TIA-1, CD20, CD3, CD103) generally clustered separately from suppressor markers/subsets (e.g., FoxP3fl PD-1fl, IDO-1, PD-L1flCD68fl) in the epithelial compartment. CD20+ B cells were significantly associated with OS (*p* = 0.03), whereas no significant association between OS and the density of CD3+ T cells or CD8+ T cells was found (*p* = 0.66 and *p* = 0.94, respectively). Thus, except for CD20+ B cells, post-NACT TIL patterns (CD3, CD8, FoxP3, and PD-1) showed a limited association with patient survival, and the increase in TILs observed after NACT was generally restricted to cases that were positive for TILs at baseline. The authors concluded that TIL-negative tumors tended to remain TIL-negative after NACT, implying that assessing TILs in pre-treatment biopsies may help to identify “immunologically inert” tumors that are unlikely to respond to immunotherapy approaches.

Similarly, Leary et al. confirmed that CD8+ ieTILs increased significantly after NACT (*p* = 0.022) but found no significant difference in median CD4+ or Foxp3+ ieTIL scores before and after NACT. Instead, the authors highlighted an important ratio result: the median of CD8+/Foxp3+, CD3+/Foxp3+, and CD4+/FOXP3+ ratios increased significantly after NACT in ieTILs (*p* = 0.0007), suggesting that NACT shifted the balance towards effector versus suppressor T-cells. No significant correlation with PFS and OS was observed. These findings argue for a specific differential effect of NACT on recruitment or expansion of conventional and activated T cells on one side and Tregs on the other side [29].

### 3.3. PD-1, PD-L1 Expression and Other Cytokine Expression

Eight studies focused on the analysis of PD-1 and PD-L1 expression and/or other immune checkpoint targets and immune populations (Table 4).

Polcher et al. showed that the mean number of granzyme B+ cells was significantly enhanced after NACT as compared to baseline (granzyme B: 2.5-fold). Similarly, granzyme B/Foxp3+ lymphocyte cell ratios were significantly increased after NACT (granzyme B/Foxp3+: 2.4-fold increase) [23].

Differently to T cell infiltration alone (CD8+), patients with high granzyme B+ infiltration (>4.5/HPF) showed, as compared to those with low cell density, a tendency for improved PFS (median 14.53 vs. 11.87 months, respectively) but not for OS. Similarly, high granzyme B+/Foxp3+ cell density ratio after NAC (>1.15) correlated significantly with extended PFS compared to low ratios (median 17.88 vs. 11.24 months, respectively). The authors concluded that NACT elicited an immunologic profile in which low immunosuppressive Foxp3+ infiltration and elevated numbers of activated granzyme B+ cells were significantly associated with PFS, suggesting a clear contribution of an active immune effector TME to improved clinical outcome [23].

Bohm et al. subsequently showed that PD-L1 protein was significantly increased after NACT compared to baseline [24]. They underlined that the potential enhancement of host antitumor immune response and reduction in mediators of cancer-related inflammation was tempered by the fact that levels of the immune checkpoint molecules PD-1 and CTLA4 on CD4+ and CD8+ T cells remained high after NACT. The authors found that good responder patients with samples scored as CRS3 had significantly improved progression-free survival (*p* = 0.002) and OS (*p* = 0.03) compared with “poor responder” patients whose biopsies scored as CRS2 [24].

Lo et al. demonstrated that IDO-1 and FoxP3+ were expressed by tumor epithelium and/or infiltrating cells in 100% of pre-NACT samples and showed no significant change in frequency or density after NACT [25]. On the contrary, iePD-1+ TILs were present in 88% of pre-NACT samples (0–7.5/100 tumor cells) and showed a marked increase after NACT, from a median of 1.2 to 1.8 cells/100 tumor cells (*p* = 0.012). To identify the cell types expressing PD-1, they performed four-color IHC, and they identified three major PD-1+ TILs subsets: PD-1+CD8−, PD-1+CD8+, and PD-1+FoxP3+ TILs. When considered individually, none of these three TILs subsets increased significantly following NACT; therefore, the overall increase in PD-1+ TILs might reflect smaller increases in all three subsets. PD-L1 was expressed in 80% of untreated tumors and showed a patchy staining pattern. Using two-color IHC, the authors found that PD-L1+ cells were a mixture of CD68+ macrophages and CD68− cells, with the latter cells comprising a mixture of other infiltrating immune cells and tumor cells. Similar to IDO-1+ cells and Tregs, PD-L1+ cells showed no significant increase following NACT. This was true for both the CD68+PD-L1+ and CD68−PD-L1+ subsets. Mesnage et al. confirmed the previous data by Bohm et al. The authors showed that the proportion of PD-L1+ immune cells indeed increased from 30% pre-NACT to 63% post-NACT (*p* = 0.04); more importantly, 63% of those PD-L1− at diagnosis became positive after NACT. In contrast to sTILs, but similar to ieTILs, PD-L1 expression was not found to be associated with survival.

On the contrary, Kim et al. evaluated the changes in PD-L1 expression after NACT: of 37 matched samples available for PD-L1 expression, PD-L1 expression remained unchanged in 18 (48.7%) patients, declined in 6 (16.2), and increased in 13 (35.1%). High PD-L1 expression in the tumor cells did not show any significant impact on either PFS (*p* = 0.764) or OS (*p* = 0.640) [27]. Similarly, PD-L1 expression on immune cells did not show any significant impact on either PFS (*p* = 0.906) or OS (*p* = 0.482).

In 2020, Chen et al. confirmed this trend, finding tumor PD-L1+ expression in 50% of OCs after NACT compared to 19.4% in treatment-naive tumors (*p* = 0.00407) [28]. Patients with high-stage OC who had PD-L1+ tumors had significantly better PFS than those who had PD-L1− tumors (28-month median in the PD-L1+ subgroup vs. 19.5-month median in the PD-L1− subgroup, *p* = 0.04). Although just shy of statistical significance at a 0.05 threshold, a similar trend was observed in the OS analysis. Interestingly, no significant difference in PD-L1 expression was detected in tumors from patients with germline BRCA1/2 mutations compared with germline mutation-negative tumors [28].

More recently, Blanc-Durand et al. found that OC tumors expressed significantly higher levels of TIM3+ (76%) than PD-L1+ (28%), IDO+ (22%), or LAG3+ (17%) [30]. When present, IDO and LAG3 were only detected on immune cells, whereas PD-L1 and TIM3 were also expressed on a minority of tumor cells. The expression of PD-L1 and TIM3 was less prevalent on tumoral than immune cells (5% vs. 28% for PD-L1 and 13 vs. 76% for TIM3). NACT had a significant impact on immune co-regulator expression, with over 70% of patients showing a change in immune protein levels: PD-L1+ tumors increased from 23% to 39% after NACT (*p* = 0.01), and LAG3+ tumors increased from 9% to 25% (*p* = 0.04), with no significant difference in the proportion of TIM3- or IDO-positive tumors. In the pre-NACT context, the authors described a positive correlation between sTILs and PD-L1 expression but none between IDO, LAG3, or TIM3 expression. In the post-NACT context, a strong correlation between sTILs and PD-L1 expression was confirmed, together with TIM3 expression and LAG3 expression. Finally, no correlation between post-NACT IDO expression and sTIL content was observed. OS and PFS were not correlated with immune checkpoint expression (TIM3, PDL1, IDO, and LAG3). Similarly, inhibitory molecule co-expression and a switch from negative to positive biomarkers or vice versa were not prognostic either [30].

Lee et al. compared PD-L1 expression in samples before and after NACT and found that in 25.2% of OCs, the initially low PD-L1 expression increased to high levels, and the degree of PD-L1 expression significantly increased after NACT (*p* = 0.003) compared to baseline [31]. The correlation between changes in Foxp3+ and the TIL level was observed with an immunostaining analysis in matched samples before and after NACT. Foxp3+ T cells were positively correlated with increasing TILs (Foxp3+: *p* = 0.003) [31]. A high expression of PD-L1 pre-NACT was associated with a significant improvement in PFS (*p* = 0.0011) and OS (*p* = 0.023). Similar results were observed for TILs; in particular, a high level of TILs pre-NACT was associated with a significantly improved PFS (*p* = 0.039) and a better OS trend (*p* = 0.077). Following this analysis, the authors compared the survival outcomes of patients with increased PD-L1 expression and TIL density to assess the prognostic impact of Delta PD-L1/TIL density for PFS and OS. No statistically significant difference in survival outcome with increased PD-L1 expression was observed. However, the PFS (*p* = 0.01) and OS (*p* = 0.027) were significantly lower in patients with increased TIL density after NACT.

### 3.4. Discussion

Neoadjuvant immunotherapy, or neoadjuvant chemo-immunotherapy, has given excellent outcomes in a variety of solid tumors, with improved response rates compared to late setting. Given that NACT with carboplatin-paclitaxel is also being used to treat an increasing number of patients with advanced OC [3,32], there has been a substantial scientific effort to try to increase the response rate of NACT with combinatorial treatments.

A deeper understanding of the evolution of immune cells before and after NACT would be beneficial in understanding the impact of NACT in the TME and potentially using this information to select individuals who could benefit from NACT+ICI combinations.

The immunophenotype (based on CD8+ infiltration) has been largely proposed as a simple way to possibly identify patients suitable for ICI treatment, but currently, no biomarker has proven to be useful for patient selection in OC.

In our review, we reported a varying effect of NACT on TME as well as a variable impact on PFS and OS.

The majority of trials reported a significant increase in s/ieTIL and CD8+ subpopulations with NACT [23,24,25,26,27,29,31], but this was only occasionally associated with an improvement in PFS and OS [26,31].

These findings imply that chemotherapy is shown to increase pre-existing TIL infiltration. On the other hand, some studies have found no relief in major immune suppressive mechanisms, or no considerable prognostic advantage was given in PFS and OS to OC patients, which might indicate that not only tumoral infiltration but also cell functionality are determinant factors to take into consideration to achieve a successful antitumor effect [24,25,33].

In fact, the lack of prognostic benefit associated with increased CD8+ TILs in patients after NACT could be explained by the concomitant upregulation of co-inhibitory molecules, or this could be because paclitaxel impairs the expansion of responsive immune cells caused by atezolizumab [23,25,27,29,34].

We found that most patients who were PD-L1-negative before NACT become PD-L1-positive after NACT, and that patients with high sTILs post-chemotherapy were significantly more likely to be PD-L1-positive [24,26,28,30,31]. This finding suggested an activation of an immunosuppressive mechanism as a regulatory response to immune stimulation during chemotherapy.

These results are consistent with the existing OC literature, in which the cooperation between chemotherapy and the immune system is still debated, given the poor outcomes of ICI–chemotherapy combinations in previous clinical trials [35,36]. Indeed, NACT has been found in site-matched samples to mainly enhance NK cells and TCR clonality, but its effect on CD8+ is less clear [24,25,37].

Sanchez et al. showed that OC TME is intrinsically heterogeneous within patients and within tumors, posing an important barrier for the successful application of immunotherapies. The induced immunogenicity following NACT was only unmasked after taking into account the TME heterogeneity, which acts as a confounding variable [38]. Instead, a significant increase in granzyme B+ [23] and PD-1 [25] after NACT was seen in a limited study, where the authors also demonstrated a significant increase in granzyme B+ T-cells after NACT, which was associated with an improvement in PFS [23]. PD-1 is a molecule expressed on T-cells, blocking their entry into the cell cycle and the production of cytokines [39], and its ligand, PDL1, causes apoptosis of T-cells upon up regulation [40] and helps the tumor evade immune destruction [41]. Similarly, they highlighted that an increase in the granzyme B1/FoxP3 ratio was positively associated with PFS [23].

Only a decrease in FoxP3+ T lymphocytes post-NACT was correlated with improved prognosis (PFS and OS) [23], suggesting that the reduction in regulatory T-cells caused by NACT may alleviate the immunosuppressive TME. However, additional research is required to corroborate these preliminary findings.

Understanding the intricate interplay between NACT and the TME in OC is challenging due to several factors. First of all, obtaining tumor samples (ideally, multiple sites within the same individual) is difficult, thus resulting in the inherent tumor heterogeneity in terms of histology and metastatic deposits. Second, clinical variables include BRCA/HRD status, the NACT schema received, and the number of cycles. Finally, no standard approach and cut-off currently exists to evaluate TILs, and the difference between tumor and stroma is poorly defined.

### Combination of Immune Checkpoint Inhibitors and Chemotherapy

The increase in TILs following NACT offers significant evidence for combining ICIs with chemotherapy. However, the results of published clinical trials have been discouraging. The phase III randomized trial of JAVELIN-100 (anti-PD-L1 avelumab with platinum-based chemotherapy versus chemotherapy alone) was prematurely terminated for the negative results at the interim analysis [36]. Similarly, in the phase III JAVELIN-200 study, the combination of doxorubicin with avelumab showed no benefit in PFS and OS compared to doxorubicin alone in platinum-resistant patients [35]. The IMAGYN050/GOG3015/ENGOT-OV39 randomized phase III study evaluating anti-PD-L1 atezolizumab with carboplatin-paclitaxel did not demonstrate any prognostic advantage of the ICIs–chemotherapy combination to chemotherapy alone. Only a subgroup analysis in patients with PD-L1 expression on ≥5% of immune cells evaluating the effect of atezolizumab on PFS suggested a potential benefit from atezolizumab [41].

Recently, the ATALANTE/ENGOT-ov29 randomized phase III trial included 614 patients with recurrent platinum-sensitive OC, having undergone one to two prior lines of chemotherapy and a progression-free interval exceeding 6 months. Participants were randomly assigned to receive either atezolizumab or a placebo for a maximum of 24 months, alongside bevacizumab and six cycles of chemotherapy doublet. The study did not achieve its primary endpoint (PFS) in the intention-to-treat group or in those positive for PD-L1 expression. Nevertheless, subgroup analysis revealed that patients negative for CD8/PD-L1 and with wild-type BRCA status, who had not previously received bevacizumab, exhibited a favorable hazard ratio with the combination of atezolizumab/bevacizumab. This suggests that bevacizumab might enhance efficacy against tumors that lack inherent immunogenicity [42]. Additionally, the topic is becoming extremely important. Especially considering the moving of ICIs in the NACT setting, further and more rigorous research is needed to uncover TME features induced by neoadjuvant treatment. For example, an ongoing trial is evaluating the benefit of neoadjuvant durvalumab (anti-PD-L1) and chemotherapy in inoperable patients (IneOV) [42]. According to preliminary results, the combination achieved an overall macroscopic complete resection rate of 70% after six cycles of NACT [43].

Other trials evaluating different ICIs in combination with NACT are currently ongoing.

The NCT05430906 trial aims to evaluate the efficacy and safety of AK104 (a bispecific antibody targeting PD-1 and CTLA-4) combined with chemotherapy as neoadjuvant treatment for advanced OC. Similarly, NCT04815408 aims to validate the efficacy and safety of anti-PD-1 (Tislelizumab) in combination with NACT.

These ongoing trials do not take into consideration the tumor-immune phenotype or any TME biomarker before beginning combination therapy.

## 4. Conclusions

Translational research to describe the TME prior to initiating ICIs+NACT and the impact of treatment on TME will be useful in identifying patients who may benefit from combination therapy.

In fact, this knowledge is critical to identify reliable predictive biomarkers and advance the field of immunotherapy in this malignancy, enabling greater treatment sophistication and hopefully providing long-lasting durable responses.

Standardization of measurements to determine the density, location, and heterogeneity of TILs throughout tumor tissue, ideally in multiple biopsies from the same patient, will partially address the genetic complexity and immunological OC heterogeneity.

In addition, it is recommended to incorporate a research outlook to evaluate the impact of chemotherapy on TILs and to track TIL dynamics throughout treatment. Investigations into the interplay between chemotherapy regimens and immune cell subpopulation immunophenotypes are also needed to determine how to select patients eligible for ICIs in the NACT setting. Moreover, exploring novel techniques such as single-cell sequencing and spatial profiling may offer deeper insights into the intricate interactions within the tumor microenvironment post-chemotherapy.

## Figures and Tables

**Table 1:** Study and patient characteristics overview.

	Study Design	Number of Patients	Number of Patients with Paired Pre- and Post-NACT	Histotype	Site of Origin	TILs Assessment	Type of CHT	Number of Cycles	FU
Polcher et al. 2010 [23]	Prospective	93	30	Serous/serous-papillary High-grade endometrioid (EC)	Intraperitoneal biopsies	Hematoxylin staining (HS) Immunohistochemistry (IHC)	Platinum-/ taxane-based NAC	3 cycles	24 months
Bohm et al. 2016 [24]	Prospective	60	54	HGSC	Omental biopsies and plasma samples	IHC, flow cytometry (FC), electrochemiluminescence assays, and RNA sequencing (RS)	Taxane/platinum combinations	3 or >4	NA
Lo et al. 2016 [25]	Retrospective	150	26 (in 18 patients, paired anatomic extra-pelvic biopsies were available)	HGSC	Omentum, pelvic site, uterus, colon, abdomen, cul-de-sac (paired only extra-pelvic biopsies)	IHC (Aperio, Pannoramic MIDI, Vectra)	Platinum-/ taxane-based NAC	A mean of 4 cycles	NA
Mesnage et al. 2016 [26]	Retrospective	150	83	High-grade serous (HGSC), EC, clear cell (CC), low-grade serous (LGSC), mucinous carcinoma (MC)	NA	Hematoxylin and eosin staining (HES) IHC	Carboplatin and paclitaxel	Average 4 cycles	52 months
Kim et al. 2018 [27]	Retrospective	266	76	HGSC	Omental biopsies	HES, IHC (Ventana XT automated stainer)	Taxane/platinum combinations	A median of 3 cycles (range, 2–6 cycles)	Median: 30 months
Chen et al. 2020 [28]	Retrospective	189	15	HGSC, CC, EC, MC	NA	HES, IHC (Aperio ScanScope AT Turbo scanner)	Platinum-based treatment	N/A	37 months
Leary et al. 2020 [29]	Retrospective	150	53	HGSC, EC, CC, other high-grade, LGSC, MC	NA	HES, IHC	Platinum and paclitaxel	Mean number of cycles: 4	Median: 80 months
Félix Blanc-Durand et al. 2020 [30]	Retrospective	98	63	HGSC, LGSC, mixed, poorly differentiated, EC, CC, MC	NA	HES, IHC (Ventana XT automated stainer)	Platinum-based treatment	N/A	N/A
Lee et al. 2022 [31]	Retrospective	147	147	HGSC	NA	HES, IHC (Ventana XT automated stainer), whole-transcriptome sequencing (WTS)	Platinum-based chemotherapy	Median of 3 cycles (range, 3–4 cycles)	Median: 28.2 months

**Table 2:** Stromal TILs (sTILs): evidence suggests an overall increase in sTILs following neoadjuvant chemotherapy (NACT), but with highly variable individual patient responses and significant prognostic implications for progression-free survival (PFS).

Stromal	Prognostic Association Pre-NACT	Prognostic Association Post-NACT	Study
TILs	PFS increase with ↑	PFS increase with ↑	Mesnage et al. [26]
	None	None	Kim et al. [27]
	PFS increase with ↑ OS increase with ↑	None	Lee et al. [31]
CD8+	None	None	Leary et al. [29]
	PFS increase with ↑ OS increase with ↑	None	Lee et al. [31]
CD3+	None	None	Leary et al. [29]
CD4+	None	None	Leary et al. [29]
	None	None	Bohm et al. [24]
Foxp3+	None	PFS increase with ↑ OS none	Leary et al. [29]
	PFS increase with ↑ OS increase with ↑	None	Lee et al. [31]

**Table 3:** Intraepithelial TILs (ieTILs): 33% of patients had increased intraepithelial TILs (ieTILs) following neoadjuvant chemotherapy (NACT). In contrast, low Foxp3+ cell density post-NACT was associated with longer progression-free survival and overall survival.

Tumor	Prognostic Association Pre-NACT	Prognostic Association Post-NACT	Study
TILs	None	None	Mesnage et al. [26]
CD8+	None	None	Polcher et al. [23]
	None	None	Lo et al. [25]
	None	None	Leary et al. [29]
CD3+	None	None	Lo et al. [25]
	None	None	Leary et al. [29]
CD4+	None	None	Polcher et al. [23]
	None	None	Leary et al. [29]
Foxp3+	None	PFS increase with ↓ OS increase with ↓	Polcher et al. [23]
	None	None	Lo et al. [25]
	None	None	Leary et al. [29]
CD20+	None	PFS none OS increase with ↑	Lo et al. [25]
CD8+/Foxp3+	None	None	Polcher et al. [23]
CD8+/CD4+	None	PFS none OS increase with ↑	Polcher et al. [23]

**Table 4:** PD-1 and PD-L1 expression, cytokine expression, and other immune checkpoint targets.

	Prognostic Association Pre-NACT	Prognostic Association Post-NACT	Study
Granzime B+	None	PFS increase with ↑ OS none	Polcher et al. [23]
GranzymeB/Foxp3	None	PFS increase with ↑ OS none	Polcher et al. [23]
PD-L1	None	None	Mesnage et al. [26]
	None	None	Kim et al. [27]
	None	None	Bohm et al. [24]
	None	None	Lo et al. [25]
	None	PFS increase with ↑ OS increase with ↑	Chen et al. [28]
	None	None	Félix Blanc-Durand et al. [30]
	PFS increase with ↑ OS increase with ↑	None	Lee et al. [31]
PD-1	None	None	Lo et al. [25]
IDO-1	None	None	Félix Blanc-Durand et al. [30]
TIM3+	None	None	Félix Blanc-Durand et al. [30]
LAG3+	None	None	Félix Blanc-Durand et al. [30]
